# Draft Beer Production Using a Nondomesticated *Saccharomyces cerevisiae*: Pilot‐Scale Comparison With a Commercial Brewing Yeast and Sensory Differentiation

**DOI:** 10.1155/ijfo/9534073

**Published:** 2026-11-10

**Authors:** Raúl Alan Velázquez-García, Erika Yanet Tapia-García, Fortunata Santoyo-Tepole, Jorge Oscar Rivera-Hernández, Guadalupe Guerra-Sánchez, Dario Rafael Olicón-Hernández

**Affiliations:** ^1^ Laboratory of Fungal Biochemistry and Biotechnology Department of Microbiology National School of Biological Sciences National Polytechnic Institute Mexico City Mexico ipn.mx; ^2^ Department of Nutrition CICS-MA National Polytechnic Institute Mexico City Mexico ipn.mx; ^3^ Department of Research National School of Biological Sciences National Polytechnic Institute Mexico City Mexico ipn.mx

**Keywords:** craft beer, nondomesticated yeast, pilot-scale fermentation, *Saccharomyces cerevisiae*, sensory analysis

## Abstract

**Background:**

The brewing industry increasingly demands alternative starter cultures capable of maintaining process reliability while generating differentiated sensory profiles. However, the industrial application of nondomesticated yeasts depends on their ability to perform competitively against established commercial strains, particularly when they originate from environments unrelated to beer production. Naturally, fermented ecosystems represent potential reservoirs of *Saccharomyces cerevisiae* diversity with unexplored value for beverage fermentation.

**Aim:**

This study is aimed at functionally screening indigenous yeast isolates obtained from a spontaneous nonbrewing fermentation system and evaluate their suitability for draft beer production through pilot‐scale comparison with a commercial yeast.

**Methods:**

Ten yeast isolates were recovered and screened for fermentative capacity using Durham tube assays. Three strains (A2, A4, and A5) showing the broadest substrate utilization were selected for further characterization. Scanning electron microscopy and internal transcribed spacer sequencing identified all three isolates as *S. cerevisiae*. Fermentation performance was evaluated at laboratory scale and subsequently validated in 100 L pilot‐scale brewing trials against the commercial ale strain US‐05. Descriptive sensory analysis was performed.

**Major Findings:**

Isolate A2 showed the highest ethanol yield (0.669 g ethanol/g substrate), the greatest attenuation (34.30%), and the highest final alcohol content (3.0%). During 100 L fermentation, A2 maintained stable process performance, reaching 3.8% alcohol by volume and 50.66% attenuation, values comparable with those obtained with US‐05 despite originating from a nonbrewing ecosystem. Sensory analysis showed that beer fermented with A2 had stronger phenolic aroma, phenolic flavor, and sweetness, whereas the commercial strain produced higher bitterness and malt character. Overall acceptance did not differ significantly between beers.

**Scientific or Industrial Implications:**

These findings demonstrate that nondomesticated yeasts recovered from nonbrewing fermentation systems can successfully compete with commercial brewing starters at pilot scale while generating differentiated products.

## 1. Introduction

The global brewing industry has undergone a sustained transition toward product diversification, largely driven by consumer demand for beers with novel sensory attributes, greater complexity, and differentiated identity [1, 2]. This trend has increased interest in alternative microbial resources capable of generating new flavor profiles while maintaining the technological reliability required in modern brewing operations [3]. Although industrial beer production still depends mainly on domesticated lineages of *Saccharomyces cerevisiae* and *Saccharomyces pastorianus*, increasing evidence indicates that substantial unexplored diversity remains within the genus *Saccharomyces*, representing an important opportunity for innovation in both sensory quality and fermentation performance [4, 5].

Recent studies have shown that intraspecific diversity within *S. cerevisiae* can produce marked differences in fermentation kinetics, stress tolerance, flocculation behavior, and the synthesis of aroma‐active compounds such as esters, higher alcohols, and organic acids. These phenotypic traits are frequently linked to the ecological origin and evolutionary history of each strain, which shape metabolic regulation and adaptive responses. Consequently, the selection of novel *Saccharomyces* strains from nonindustrial environments has emerged as an attractive strategy to expand the functional repertoire of brewing yeasts while preserving compatibility with conventional brewing processes [6–8].

Despite this potential, the practical use of newly isolated *Saccharomyces* strains remains limited. Wild or nondomesticated yeasts often show slower sugar utilization, lower ethanol tolerance, unstable flocculation, or inconsistent metabolite production under brewery conditions. In addition, many strains that perform adequately at laboratory scale fail during pilot or industrial fermentation, where oxygen transfer, hydrostatic pressure, heat dissipation, and sedimentation dynamics differ substantially from small‐scale systems. Therefore, identifying nondomesticated strains capable of competing with commercial starters while simultaneously delivering differentiated sensory profiles remains a major challenge in brewing biotechnology [8–10].

Naturally, fermented systems constitute dynamic ecological niches in which microbial populations are continuously exposed to selective pressures such as nutrient limitation, osmotic stress, ethanol accumulation, and interspecies competition. These conditions may favor the emergence of yeast strains with enhanced robustness, metabolic flexibility, and stress resistance. For this reason, spontaneous fermentations are increasingly recognized as valuable sources of *Saccharomyces* diversity, particularly lineages that have not undergone the intensive domestication associated with brewing strains [4, 9].

Pulque, a spontaneously fermented agave‐based beverage, is one such ecosystem and is characterized by the coexistence of yeasts and bacteria in a carbohydrate‐rich matrix. During fermentation, fluctuations in substrate composition and microbial interactions may select for *Saccharomyces* populations with distinctive physiological traits. Although pulque has long been recognized for its microbial diversity, its potential as a source of nonbrewing *Saccharomyces* strains capable of adaptation to beer fermentation remains insufficiently explored [11, 12].

Compared with strategies based on non‐*Saccharomyces* yeasts, the exploration of *Saccharomyces* diversity from alternative fermentation environments offers a more direct route toward industrial application. Because these strains belong to the same species most used in brewing, they are more likely to retain efficient sugar utilization, ethanol productivity, and process compatibility, while still contributing novel sensory traits derived from their distinct ecological background. Thus, bioprospecting nondomesticated *Saccharomyces* from spontaneous fermentations may provide starter cultures capable of generating differentiated beer products without requiring radical process modifications [13, 14].

In this context, the present study is aimed at isolating and screening yeast strains from a spontaneous nonbrewing fermentation system, evaluate their fermentation performance in draft beer production, and compare the best‐performing isolate with a commercial brewing strain under pilot‐scale conditions (100 L). Particular emphasis was placed on determining whether a nondomesticated *S. cerevisiae* could combine technological robustness with sensory differentiation, thereby contributing new alternatives for industrial and craft brewing biotechnology.

## 2. Materials and Methods

### 2.1. Reagents and Culture Media

All reagents used in this study were purchased from Merck (Darmstadt, Germany). The commercial source of specific reagents is indicated throughout the manuscript when appropriate.

A commercially available Sabouraud Dextrose Agar (SDA) medium (Merck, Darmstadt, Germany) was used for the isolation and cultivation of fungi and yeasts. According to the manufacturer′s specifications, the medium contained glucose (40 g/L), peptone (10 g/L), and agar (15 g/L). The pH was adjusted to 5.6 before sterilization by autoclaving at 121°C for 15 min at 15 psi.

### 2.2. Pulque Sampling

Fermented beverage samples were collected from four traditional pulque establishments located in Mexico City (Locations: 19.430433602122353, −99.1427475631529; 19.3613781352898, −99.15137935575656; 19.434274774915945, −99.14652870232823 and 19.458226338786293, −99.1283295446562). Both final fermented pulque produced after 48–72 h of fermentation during the autumn season, and sediment samples were obtained. The beverage samples were manually homogenized, and 50 mL aliquots were transferred into sterile Falcon tubes. Sediments were manually collected and stored in the same type of sterile tubes. All samples were transported on ice and refrigerated until analysis.

### 2.3. Yeast Isolation and Selection

Samples were homogenized and serially diluted from 10^0^ to 10^−5^ using sterile saline solution. Aliquots of 500 *μ*L from dilutions 10^−3^, 10^−4^, and 10^−5^ were spread onto SDA plates and incubated at 28°C for 18–24 h. Colonies were evaluated morphologically and microscopically using bromophenol blue staining. Colonies exhibiting typical yeast morphology were selected and subcultured on SDA plates to ensure purity. Pure isolates were preserved in cryovials containing 25% glycerol at freezing temperature.

### 2.4. Evaluation of Fermentative Capacity: Durham Test

The fermentative capacity of the isolates was determined using the Durham tube method [15]. Test tubes containing a medium composed of 3 g/L ammonium nitrate and 62.5 mL mineral salt solution supplemented with 20 g/L glucose, sucrose, or maltose were prepared. These sugars were selected because they are commonly present in brewing media. Each tube was inoculated with a loopful of yeast biomass previously grown on SDA agar for 18 h, and an inverted Durham tube was inserted. The tubes were incubated for 4 days, and CO_2_ production was monitored every 24 h by measuring gas accumulation within the Durham tube.

Gas production was qualitatively classified as high, medium, low, or null fermentative capacity using an arbitrary numerical scale: 1.0 (high), 0.5 (medium), 0.25 (low), and 0.0 (null), according to the volume of gas produced. Isolates classified as high or medium fermenters and capable of fermenting most of the tested sugars were selected for further study. All experiments were performed in triplicate. However, because the results were evaluated using an arbitrary semiquantitative scoring system, no statistical analysis was performed. Consequently, a single representative score is reported, and no standard deviation is presented, as the assigned scores do not constitute continuous quantitative data.

### 2.5. Characterization and Identification of Selected Isolates

#### 2.5.1. Scanning Electron Microscopy

Yeast cultures were fixed in 2.5% glutaraldehyde prepared in 0.1 M phosphate‐buffered saline (PBS), followed by three washing steps. Samples were dehydrated through an ethanol gradient (30%–100%) and dried by critical point drying using liquid CO_2_. Dried samples were mounted on metallic stubs using conductive carbon tape and examined using a JEOL‐5800LV scanning electron microscope operated at 15 kV [16].

#### 2.5.2. Molecular Identification

Yeasts were grown in Sabouraud broth for 18 h, and biomass was harvested by centrifugation (3000 × g, 10 min). The biomass was washed with sterile distilled water. Genomic DNA was extracted using the JetSorb DNA Extraction Kit (Genomed GmbH, Bad Oeynhausen, Germany). PCR amplification of the internal transcribed spacer (ITS) region was performed using primers ITS1 (5 ^′^‐TCCGTAGGTGAACCTGCGG‐3 ^′^) and ITS4 (5 ^′^‐TCCTCCGCTTATTGATATGC‐3 ^′^). The ITS1 and ITS4 primer pair was selected because it is one of the most widely used molecular markers for the identification of yeasts and other fungi, providing reliable amplification of the ITS1–5.8S–ITS2 region [17]. PCR products were purified using ExoSAP‐IT PCR Product Cleanup Reagent (Applied Biosystems, Thermo Fisher Scientific, Waltham, Massachusetts, United States) and sent for sequencing. Sequence identification was performed using BLAST (NCBI). Phylogenetic analysis was conducted according to our previously reported protocol. Sequences were aligned using the MUSCLE algorithm implemented in MEGA12 software. A phylogenetic tree was then reconstructed using the maximum likelihood statistical method under the Tamura–Nei three‐parameter evolutionary model [18].

### 2.6. Fermentation Kinetics at Laboratory Scale

Selected yeasts were grown on SDA plates for 18 h at 28°C. A loopful of biomass was inoculated into a 250 mL Erlenmeyer flask containing 30 mL of unhopped commercial malt wort (2‐row base malt) adjusted to 10°Brix. All °Brix adjustments described in this study were performed by dilution with sterile distilled water using the dilution equation *C*_1_ *V*_1_ = *C*_2_ *V*_2_. Cultures were incubated at 28°C with agitation at 125 rpm for 18 h.

Fermentations were carried out in 500 mL transparent borosilicate bioreactors equipped with an airlock and a stainless‐steel sampling valve at room temperature. Each reactor contained 500 mL of wort adjusted to 10°Brix and was inoculated with 30 mL of the yeast starter culture adjusted at 0.8 UDO_600nm_/mL. Fermentations were conducted under static conditions for up to 125 h. Periodic samples were collected to evaluate biomass formation, sugar consumption, and ethanol production. All experiments were performed in triplicate.

### 2.7. Analytical Methods for Laboratory‐Scale Fermentations

#### 2.7.1. Biomass Determination

Cell biomass was determined by direct counting using a Neubauer chamber. Fermentation samples of 100 *μ*L were diluted 1:10 with distilled water. Then, 10 *μ*L of the diluted sample was loaded into the chamber for cell concentration determination, considering the dilution factor and chamber volume.

#### 2.7.2. Ethanol Quantification by Gas Chromatography

Ethanol concentration was determined by gas chromatography with flame ionization detection (GC‐FID) using an Agilent 7890 system equipped with a Combi‐PAL headspace sampler. Headspace conditions were incubation temperature, 80°C; syringe temperature, 85°C; equilibration time, 15–20 min. Chromatographic separation was performed using a J&W DB‐WAXetr column (0.53 mm × 30 m, 2 *μ*m film thickness). Oven temperature was held at 40°C for 10 min, then increased at 25°C/min to 240°C and held for 1 min. Detector and injector temperatures were 250°C and 150°C, respectively. Helium was used as carrier gas at 7 mL/min, and injection volume was 200 *μ*L [19].

#### 2.7.3. Sugar Consumption

Residual sugars were estimated using a KingtenStar professional refractometer (KingtenStar, Shenzhen, China). The instrument was calibrated with distilled water. One milliliter of sample was placed on the prism surface, and °Brix values were recorded. Sugar consumption was calculated from the difference between initial and residual soluble solids [20].

### 2.8. Fermentation Kinetics at 100 L Scale

A loopful of yeast biomass from an 18 h SDA plate was inoculated into 30 mL wort adjusted to 10°Brix and incubated for 18 h at 30°C with agitation. The total culture was transferred into a 1 L flask containing 250 mL wort (10°Brix) and incubated for an additional 18 h. Cell density was measured using a Neubauer chamber.

This inoculum was transferred into a 20 L food‐grade polypropylene bioreactor containing 15 L of wort for biomass propagation at room temperature. The propagated inoculum was subsequently transferred into a 150 L stainless steel conical fermenter with a working volume of 100 L. The fermenter was equipped with a cooling jacket, sampling valve, and an airlock calibrated to 1 psi. The fermentation temperature was maintained at 20°C, and the process was carried out for 2 weeks. This fermentation period was selected according to the standard operating protocol routinely used by the craft brewery for this pilot‐scale bioreactor. Biomass formation and sugar consumption were measured using the same procedures described for the laboratory scale. Alcohol production was determined using a commercial densimeter previously validated against the gas chromatographic method described above. The determinations were performed in triplicate for each batch.

### 2.9. Beer Processing and Formulation

For the 100 L beer batches, reverse osmosis water was adjusted to pH 5.5 using phosphoric acid. Wort was prepared using 100% Pilsner 2‐row malt (Altiplano, Mexico), targeting an original gravity of 1.045 (11.25°Bx). Galaxy hops (15% alpha acids; Hop Products Australia, Tasmania, Australia) were used to achieve 32.6 IBU.

Two independent batches were produced: one fermented with the commercial yeast SafAle US‐05 (hereafter referred to as US‐05; Fermentis, Marcq‐en‐Barœul, France) and the other with the selected isolate showing the best laboratory‐scale performance. Malt was milled using a motorized roller mill. Mashing was conducted at 60°C for 1 h, followed by lautering and wort boiling for 1 h. Heating was performed using a direct‐fire butane gas burner.

Carbonation was performed at 28 psi using a Quick Carb system (Blichmann Engineering, Lafayette, Indiana, United States) in 60 L kegs containing 50 L of beer. Bottling was carried out using a manual counter‐pressure bottle filler and a hand‐operated capper.

### 2.10. Kinetic Parameters

The maximum specific growth rate (*μ*max), biomass yield on substrate (Yx/s), alcohol by volume (%ABV), and attenuation (%) were calculated according to the following equations:

(1) μmax=lnX2−lnX1t2−t1,

(2) Yx/s=X−X0S0−S,

(3) ABV%=OG−FG×131.25.

(4) Attenuation%=OG−FGOG−1×100.

where *X* is biomass concentration, *t* is time, *S*_0_ is initial substrate concentration, *S* is residual substrate concentration, *O* *G* is original gravity, and *F* *G* is final gravity.

### 2.11. Fourier‐Transform Infrared Spectroscopy (FT‐IR) Analysis of Final Product

The chemical characterization of the final beers was performed by FT‐IR. This technique was selected because it provides a rapid and nondestructive approach for beer characterization and quality assessment. Although beer is a chemically complex matrix with highly overlapping absorption bands, FT‐IR has been widely applied for the characterization of beers and for monitoring compositional changes during fermentation [21, 22]. One milliliter of each sample was directly placed on the ATR crystal of a PerkinElmer Spectrum 2000 FT‐IR spectrometer (software Version 5.31). Spectra were recorded over the 4000–400 cm^−1^ range at a resolution of 4 cm^−1^ and were used for the comparative analysis of the characteristic absorption bands of the final beer samples.

### 2.12. Sensory Analysis

#### 2.12.1. Basic Taste Recognition Test

All subjects gave their informed consent for inclusion before they participated in the study. A total of 14 panelists participated in the sensory evaluation. Panelists were initially screened according to the basic sensory method described by Watts et al. [23], which evaluates the ability to correctly recognize the four basic tastes using standard reference solutions. Taste solutions were prepared with distilled water containing sucrose (1% w/v), sodium chloride (0.2% w/v), citric acid (0.04% w/v), and quinine sulfate (0.00125% w/v). Approximately 25–30 mL of each solution was served in cups coded with random three‐digit numbers and presented in a randomized order. Participants were instructed to rinse their mouths with water between samples and to identify the predominant taste of each solution. Only panelists who correctly identified all four basic tastes were selected.

The selected panelists were subsequently trained on the definitions of each sensory attribute (supporting information available here) and on beer tasting techniques according to Mosher [24]. In addition, comparative tasting sessions using commercial beers were conducted to establish and calibrate the evaluation scale prior to the sensory analysis.

#### 2.12.2. Bitterness Ranking Test

Five quinine sulfate solutions of increasing concentration (0%–0.004%) were prepared using carbonated mineral water. Samples were coded and presented simultaneously in randomized order. Panelists ranked samples from lowest to highest bitterness intensity. A maximum of one ranking error was accepted.

#### 2.12.3. Descriptive Sensory Analysis

An agile descriptive sensory methodology was applied using a semitrained panel. Twenty‐five sensory attributes were evaluated using a 1–9 intensity scale. Only panelists who passed both screening tests were included.

Each panelist evaluated four samples: two replicates of beer fermented with the selected isolate and two replicates of beer fermented with US‐05. Samples were coded with random three‐digit numbers and served in transparent 60 mL containers in randomized order. Water was provided for palate cleansing between samples.

Data consistency was evaluated within each panelist, and inconsistent responses were excluded. Means and standard errors were calculated. Data were analyzed by two‐way ANOVA considering product and panelist as fixed factors for each sensory attribute. Tukey′s HSD test was applied when significant product effects were detected (*p* < 0.05). Effect size was estimated using eta squared (*η* ^2^). Analyses were performed using custom macros developed in Microsoft Excel (Visual Basic).

## 3. Results

### 3.1. Yeast Isolation

Ten isolates (A1–A10) showing morphological characteristics consistent with yeasts were recovered on SDA. The colonial and microscopic characteristics of these isolates are shown in Figure 1. In general, all isolates formed circular to slightly convex colonies with smooth surfaces, creamy‐white coloration, and entire margins, which are typical features of yeast growth. Differences in colony size, abundance, and growth density were observed among isolates, indicating phenotypic variability within the recovered population.

**Figure 1:**
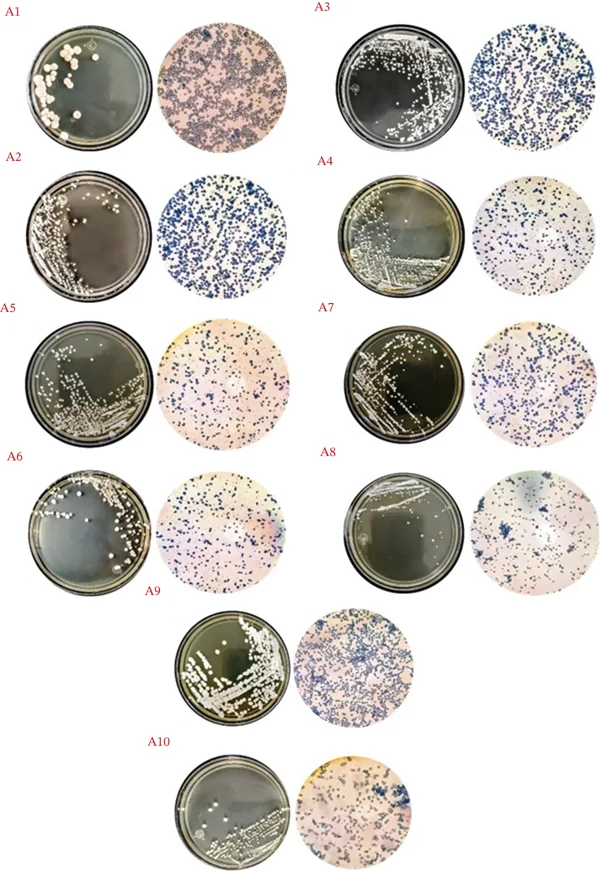
Colonial and microscopic morphology of yeast isolates (A1–A10) recovered from pulque samples. Left: colony morphology on Sabouraud Dextrose Agar (SDA). Right: microscopic morphology after staining.

Some isolates, such as A1, A6, and A9, developed larger and denser colonies, whereas A8 and A10 showed lower colony development. The remaining isolates exhibited intermediate growth with abundant and homogeneous colony distribution under the culture conditions used. Microscopic examination revealed predominantly oval to spherical cells with evident budding. Cells were observed individually, in pairs, or in small aggregates. No pseudohyphae or filamentous structures were detected in any isolate, supporting their preliminary classification as yeast.

### 3.2. Fermentative Capacity of Pulque Yeast Isolates

The fermentative performance of the 10 isolates was evaluated using the Durham tube method with glucose, sucrose, and maltose as carbon sources (Table 1). Considerable variability was observed among isolates depending on the substrate tested. In general, glucose supported the highest fermentative activity, whereas sucrose showed intermediate values and maltose was more poorly utilized.

**Table 1:** Fermentative capacity of yeast isolates determined by the Durham test using glucose, sucrose, and maltose as carbon sources.

Isolates	Fermentative capacity		
	Glucose	Sucrose	Maltose
A1	0.125	0.50	0.0625
A2	1.000	0.50	0.0625
A3	0.125	0.00	0.50
A4	1.000	0.50	0.75
A5	1.000	0.25	0.50
A6	0.00	0.00	0.0625
A7	0.50	0.00	0.125
A8	0.25	0.00	0.25
A9	0.00	0.00	0.00
A10	0.25	0.25	0.0625

*Note:* Values represent a semiquantitative score. Experiments were performed in triplicate; however, no statistical analysis or standard deviation is reported due to the arbitrary scoring system.

Among all isolates, A2, A4, and A5 were identified as the most promising candidates because they were able to ferment all three sugars. These three isolates exhibited strong glucose fermentation. Sucrose fermentation was moderate in all cases, whereas maltose utilization differed between strains: A4 showed the highest activity, A5 showed intermediate performance, and A2 displayed lower but detectable fermentation. Based on their broader substrate utilization profiles, these isolates were selected for fermentation kinetics studies.

### 3.3. Morphological and Molecular Characterization of the Selected Yeast

Scanning electron microscopy of isolates A2, A4, and A5 revealed the typical morphology of budding yeasts (Figure 2). Cells were predominantly ovoid to ellipsoidal, with sizes ranging from approximately 3–8 *μ*m. Surfaces appeared smooth and intact, and active multipolar budding with visible bud scars was frequently observed. Cells were distributed individually or in small aggregates. No relevant structural differences were detected among the three, suggesting high morphological similarity at the cellular level.

**Figure 2:**
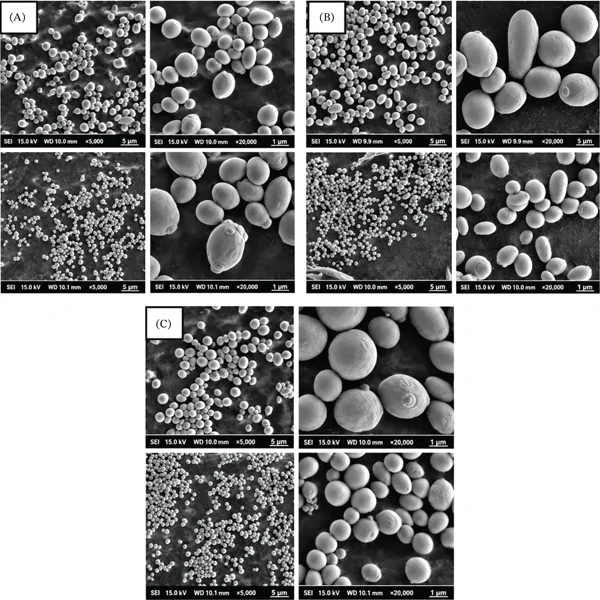
Scanning electron micrographs (SEM) of isolates (A) A2, (B) A4, and (C) A5. Images were acquired at magnifications of 500×, 1000×, 2500×, and 5000×. Scale bars are shown in each image.

Sequencing of the ITS region demonstrated high identity values with reference sequences of *S. cerevisiae*: 99.73% for isolate A5 (Access Number NCBI: PZ320974), 99.36% for A2 (Access Number NCBI: PZ320973), and 99.72% for A4 (Access Number NCBI: PZ320981). According to accepted taxonomic criteria for fungal ITS identification, these results support assignment of all three isolates to the species *S. cerevisiae*.

The phylogenetic tree constructed using the neighbor‐joining method is shown in Figure 3. All three isolates clustered within the *S. cerevisiae* clade, confirming their taxonomic identity. Although the updated topology places A2, A4, and A5 within a closely related species cluster, noticeable branch separation among the isolates still suggests the presence of intraspecific genetic divergence. Isolates A2 and A5 appeared more closely associated, whereas A4 showed a relatively more separated position within the *S. cerevisiae* group, indicating a differentiated lineage at the strain level. These differences, whereas moderate, may be biologically relevant and could help explain the contrasting fermentation behaviors observed among the isolates, particularly regarding sugar utilization, ethanol productivity, flocculation, and aroma formation.

**Figure 3:**
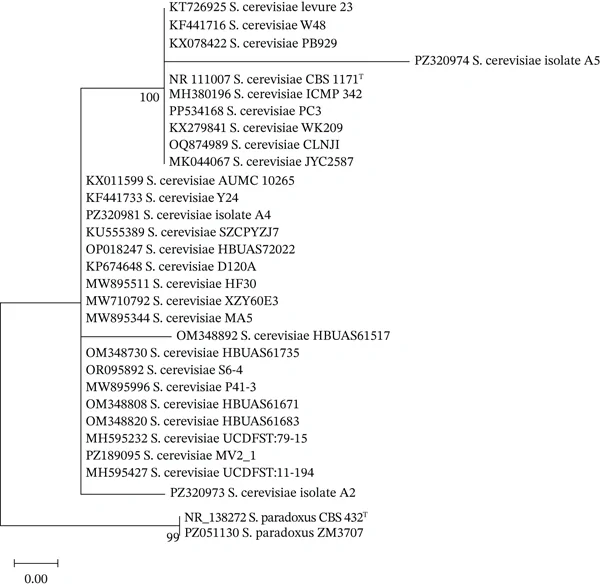
Phylogenetic tree based on ITS1 rDNA sequences of isolates A2, A4, and A5 constructed using the Neighbor‐Joining method. Multiple sequence alignment was generated with MUSCLE as available in MEGA12. Evolutionary relationships were inferred by constructing a maximum likelihood phylogeny using the Tamura–Nei model with three parameters. Reference strains marked with superscript “T” correspond to recognized type strains used for taxonomic comparison.

### 3.4. Laboratory‐Scale Fermentation Kinetics

The three selected isolates showed a similar general fermentation pattern at laboratory scale (Figure 4). Biomass increased rapidly during the first 24 h, corresponding to an exponential growth phase, followed by entry into stationary phase. Subsequently, a decline in suspended cell counts was observed, which coincided with visible sediment formation at the bottom of the bioreactor, indicating flocculation and settling behavior.

**Figure 4:**
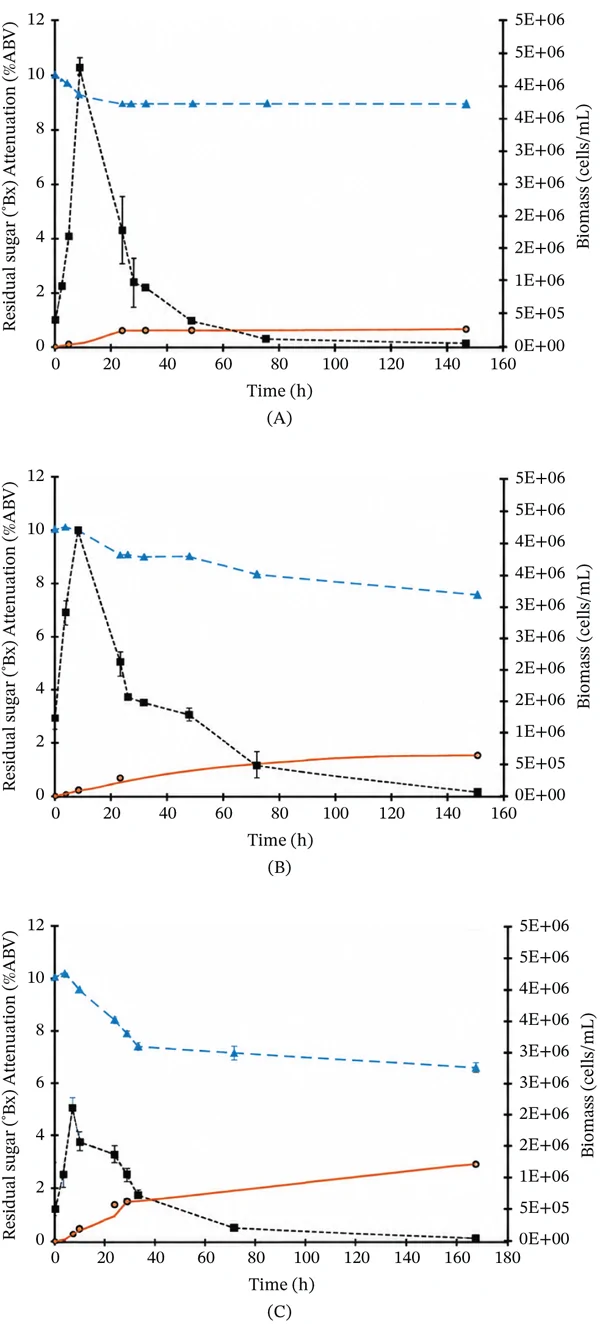
Fermentation kinetics of isolates (A) A5, (B) A4, and (C) A2 during laboratory‐scale wort fermentation. Residual soluble solids (blue lines), apparent attenuation (black lines), and biomass (red lines) were monitored throughout the fermentation. Statistical comparisons were not performed at individual time points.

Sugar concentration progressively decreased throughout fermentation, accompanied by ethanol accumulation. Although isolate A2 reached a lower maximum suspended biomass than A4 and A5, it displayed the highest sugar consumption rate, suggesting more efficient substrate conversion. The initial pH was 6.0 ± 0.1 for all three isolates. The final pH values were 5.1 ± 0.2 for A2, 5.3 ± 0.2 for A5, and 5.5 ± 0.1 for A4.

As shown in Figure 5, isolate A2 also exhibited superior ethanol production kinetics. Ethanol concentration increased continuously in all fermentations; however, A2 showed the fastest production rate and reached the highest final alcohol content.

**Figure 5:**
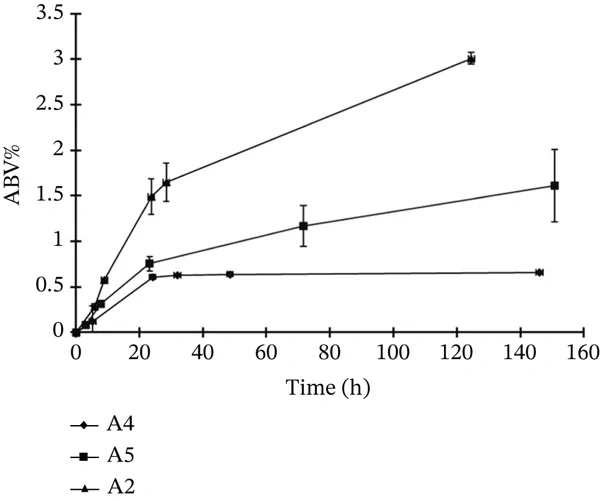
Comparative ethanol production kinetics of isolates A2, A4, and A5 during laboratory‐scale fermentation, determined by gas chromatography and expressed as %ABV over time. Statistical comparisons were not performed at individual time points.

Calculated kinetic parameters are summarized in Table 2. Isolate A2 showed the highest ethanol yield (0.669 g ethanol/g substrate), the highest attenuation (34.30%), and the highest final alcohol content (3.0% ABV). In contrast, isolates A5 and A4 exhibited slightly higher *μ*max values but lower conversion efficiency and lower attenuation, indicating less favorable overall fermentation performance. Based on these results, A2 was selected for scale‐up trials.

**Table 2:** Kinetic parameters of isolates A5, A2, and A4 during laboratory‐scale fermentation. Values represent the mean of three independent replicates.

Parameter	A5	A2	A4
*μ*max (h^−1^)	0.271 ± 0.026^a^	0.236 ± 0.05^b^	0.270 ± 0.0003^a^
Yp/s (g ethanol/g substrate)	0.508 ± 0.011^a^	0.669 ± 0.027^b^	0.521 ± 0.03^c^
Attenuation (%)	24.75 ± 2.60^a^	34.30 ± 2.08^b^	10.00 ± 0.01^c^
Alcohol content (%ABV)	1.6 ± 0.041^a^	3.0 ± 0.021^b^	0.6 ± 0.02^c^

*Note:* Different letters indicate statistically significant differences according to a one‐way ANOVA followed by Tukey′s post hoc test (*p* < 0.05), performed using SigmaPlot v.15 (Systat Software Inc., San Jose, California, United States).

### 3.5. 100 L Scale Fermentation Performance

Two 100 L batches were produced under identical brewing conditions: one fermented with isolate A2 and the other with the commercial ale yeast US‐05, an American‐style high‐fermentation strain of *S. cerevisiae*. This strain was selected because of its ease of handling and because the original brewing process had been previously standardized using this yeast.

Fermentation kinetics of isolate A2 revealed a characteristic three‐phase pattern (Figure 6A). During Phase I (0–53.3 h), cell concentration increased 22.5‐fold, from 80,000 to 1,800,000 cells/mL, whereas approximately 40% of the final ethanol concentration was achieved (1.58% ABV). In Phase II (53.3–147.1 h), biomass remained stable at approximately 1.8–1.9 million cells/mL, whereas ethanol concentration increased to 2.89% ABV. During Phase III (147.1–355.9 h), suspended biomass declined by 76.4%, corresponding to maturation and sedimentation stages, whereas residual sugars were further consumed to reach a final alcohol content of 3.8% ABV. The fermentation proceeded without visible signs of microbial contamination or sensory defects. Foam formation remained moderate and did not generate operational problems.

**Figure 6:**
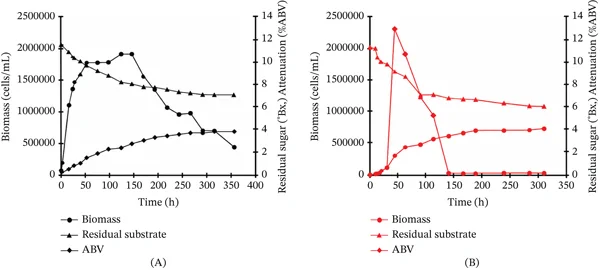
Comparative fermentation kinetics of 100 L brewing batches produced with isolate A2 and the commercial ale yeast US‐05. (A) Time‐course profile of isolate A2 showing suspended biomass (cells/mL), residual soluble solids (°Bx), and ethanol production (%ABV) during fermentation. (B) Fermentation profile of the commercial control strain SafAle US‐05 under the same brewing conditions. Statistical comparisons were not performed at individual time points.

The fermentation performance of isolate A2 at the 100 L scale was comparable with that of the commercial strain US‐05 (Table 3). Although A2 exhibited a significantly lower *μ*max than US‐05, no significant differences were observed in final attenuation or alcohol production. Both strains also showed a similar decrease in pH throughout fermentation. Biomass flocculation, however, was more pronounced in the batch fermented with the commercial strain (Figure 6).

**Table 3:** Comparison of the main fermentation parameters of isolate A2 and the commercial brewing strain SafAle US‐05 during pilot‐scale (100 L) beer fermentation.

Parameter	A2	US‐05
*μ*max (h^−1^)	0.0573 ± 0.001^a^	0.233 ± 0.002^b^
Final attenuation (%)	50.66 ± 1.2^a^	46.09 ± 2.3^a^
Alcohol (%ABV)	3.80 ± 0.53^a^	4.20 ± 0.12^a^
Initial pH	6.3 ± 0.1^a^	5.8 ± 0.1^a^
Final pH	5.0 ± 0.2^a^	4.7 ± 0.3^a^

*Note:* Different letters indicate statistically significant differences according to a one‐way ANOVA followed by Tukey′s post hoc test (*p* < 0.05), performed using SigmaPlot v.15 (Systat software Inc., San Jose, California, United States).

### 3.6. FT‐IR Analysis of Final Product

FT‐IR spectra comparing the base wort and the final fermented beers are presented in Figure 7. Fermented samples showed a clear reduction in carbohydrate‐associated signals (1200 cm^−1^), together with the appearance of new bands associated with fermentation metabolites (900 cm^−1^). These changes were mainly associated with C–O stretching vibrations of alcohols and carbohydrates.

**Figure 7:**
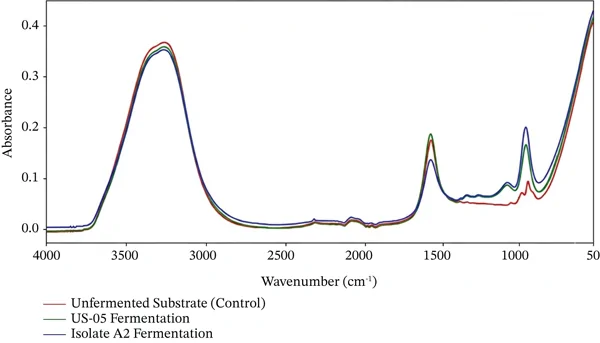
FT‐IR spectra of base wort, beer fermented with isolate A2, and beer fermented with the commercial strain US‐05.

Compared with US‐05, beer fermented with isolate A2 showed greater signal intensity around 1040 cm^−1^, suggesting a slightly higher concentration of secondary metabolites such as polyhydroxylated compounds, glycerol, or higher alcohol derivatives. Conversely, the commercial strain showed stronger signals near 1450 cm^−1^ and 1100 cm^−1^, typically associated with standard fermentation metabolites and glycerol‐related compounds [21, 22]. In contrast, the unfermented substrate control did not show these modifications.

### 3.7. Sensory Evaluation of Final Product

Initially, 14 candidates volunteered for panel participation. All passed both the basic taste recognition and bitterness ranking tests.

An intrapanelist repeatability analysis was then performed based on differences between replicate evaluations. Panelists showing more than 20% of attributes with differences greater than three points on the 9‐point scale were considered inconsistent. Using an operational exclusion threshold of 25%, three panelists.

Mean scores for the 25 sensory attributes are presented in Figure S1 of Supporting Information. In general, both beers showed similar sensory performance, with only a limited number of attributes differing significantly. The beer produced with isolate A2 showed higher sweetness and stronger phenolic character than the US‐05 control. Two‐way ANOVA (*α* = 0.05) confirmed significant product effects mainly for flavor‐ and aroma‐related attributes, including phenolic aroma, phenolic flavor, acidic taste, sweet taste, bitter taste, malt character, and astringency. Tukey′s post hoc test confirmed these differences. Effect size analysis (*η* ^2^) showed moderate to high contributions of product type to these attributes, especially phenolic, sweet, and acidic descriptors (Figure 8). In contrast, visual descriptors such as color, turbidity, foam, and overall acceptance did not differ significantly between samples. These results indicate that product differentiation was driven primarily by flavor and aroma rather than appearance or global liking.

**Figure 8:**
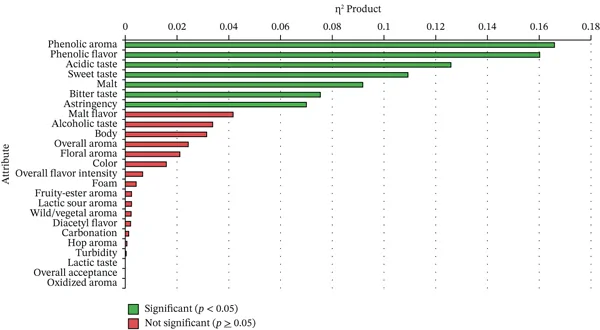
Effect size analysis (*η* ^2^) of product type on the evaluated sensory attributes for beers fermented with isolate A2 and the commercial strain US‐05. Bars indicate the relative contribution of sample identity to each descriptor based on two‐way ANOVA.

The radial sensory plot (Figure 9) further illustrates the distinct profiles of both beers. The A2 beer was characterized by higher phenolic aroma, phenolic flavor, and sweet taste, whereas the control beer tended toward greater bitterness and malt intensity. According to panel comments, the phenolic character associated with A2 was mainly described as clove‐like.

**Figure 9:**
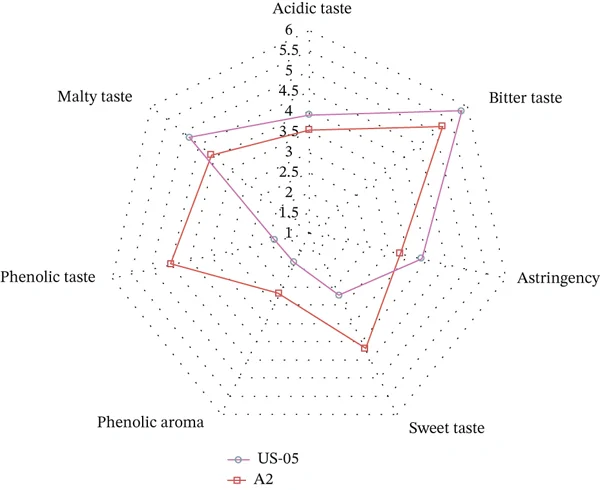
Radial sensory profile comparing beer fermented with isolate A2 and the commercial control strain US‐05 for the attributes showing the greatest product effect.

## 4. Discussion

The present study demonstrates that spontaneous fermented beverages can serve as valuable reservoirs of brewing‐relevant *S. cerevisiae* diversity, with performance comparable with that of commercial yeasts. Rather than focusing solely on microbial occurrence, the practical importance of these ecosystems lies in their potential to provide strains able to generate differentiated products while maintaining technological performance [25]. Pulque has historically been recognized as a microbiologically complex fermentation system, and its value as a source of strains for biotechnological applications has gained increasing attention in recent years. For example, Reyes‐Vidal et al. isolated several yeasts from artisanal pulque and selected strain CR4 due to its broad carbohydrate assimilation profile, including glucose, xylose, arabinose, sucrose, maltose, and lactose. That isolate was successfully applied in a microbial fuel cell using agro‐industrial wastewater as substrate, demonstrating that pulque‐derived yeasts may possess metabolic versatility and tolerance to challenging fermentation environments [26].

In the present work, 10 yeast isolates were recovered, and three candidates showed fermentative potential for implementation in a brewing process using a substrate markedly different from their original ecological environment. This cross‐environment adaptation is particularly relevant because the successful transfer of yeasts from nonbrewing systems into beer production represents an attractive route for starter innovation. Similar observations have been reported for strains originating from other fermentation sectors. For instance, Lorca Mandujano et al. isolated the *S. cerevisiae* strain LBGA‐287 from a Brazilian bioethanol fermentation process and demonstrated its suitability for beer production. In pilot‐scale fermentations of 45 L Belgian‐style ale beer, LBGA‐287 showed fermentation behavior comparable with the commercial control strain, with efficient sugar attenuation, similar pH reduction, and a final ethanol concentration superior to the reference yeast. Sensory trials involving 70 evaluators (63 consumers and 7 trained sommeliers) showed 24% higher overall acceptance for the beer produced with LBGA‐287, which was described as having improved body, lower acidity, and distinctive clove‐ and cinnamon‐like aromatic notes [27].

Although all three strains clustered within the *S. cerevisiae* lineage, the phylogenetic separation suggests meaningful intraspecific divergence. Phenotypic variation in *S. cerevisiae* has frequently been associated with ecological origin, domestication history, and adaptation to distinct fermentation niches. Gallone et al. analyzed 157 industrial *S. cerevisiae* strains and demonstrated that modern industrial yeasts are separated into five major lineages, including two independent beer clades (Beer 1 and Beer 2), each with distinct genetic and phenotypic traits. Their study showed that Beer 1 strains typically produced 7.5%–10% v/v ethanol, whereas strains associated with sake, spirits, wine, and bioethanol reached up to 14.5% v/v, reflecting strong niche‐dependent metabolic specialization [28].

The same authors also reported lineage‐specific adaptations directly relevant to brewing performance. Beer strains showed improved utilization of wort sugars such as maltotriose through amplification or modification of *MAL* transporter genes, whereas many wine strains had partially lost these loci. In addition, several brewing strains accumulated loss‐of‐function mutations in *PAD1* and *FDC1*, reducing the production of 4‐vinyl guaiacol, a phenolic note undesirable in many beer styles [28]. These observations demonstrate that relatively close phylogenetic groups within *S. cerevisiae* can differ markedly in attenuation capacity, aroma production, and industrial robustness. Therefore, the phylogenetic separation observed for A2, A4, and A5 may partially explain their contrasting fermentation behaviors. Although all three isolates belong to the same species, microevolutionary divergence may have generated differences in sugar assimilation efficiency, ethanol productivity, flocculation behavior, and secondary metabolite formation. Whole‐genome sequencing would be particularly valuable to identify variations in loci associated with brewing performance, such as *MAL* genes involved in maltose/maltotriose uptake, *FLO* genes related to flocculation, and *PAD1/FDC1* genes associated with phenolic aroma production. Such analyses would help explain why isolate A2 outperformed A4 and A5 despite their close phylogenetic relationship.

The carbohydrate screening assay revealed substantial differences in substrate utilization. Glucose supported the strongest fermentative responses, whereas maltose utilization was generally limited. This pattern is consistent with the ecology of pulque fermentation, where agave sap is dominated by sucrose, glucose, and fructose rather than maltose‐containing substrates. In such environments, selective pressure to maintain highly efficient maltose transport and catabolic systems is likely reduced. The lower performance observed for several isolates may therefore reflect partial downregulation or reduced competitiveness of the *MAL* loci commonly associated with maltose assimilation in brewing strains [7, 29]. This interpretation is particularly relevant because maltose is the principal fermentable sugar in brewer′s wort, meaning that ecological adaptation can directly influence brewing suitability.

An important observation was that A2 did not exhibit the highest *μ*max at laboratory scale, yet it produced the highest ethanol concentration and attenuation. This confirms that maximum growth rate should not be interpreted as the sole predictor of brewing performance. Rapid biomass formation may reflect allocation of carbon toward cell proliferation rather than ethanol generation. In contrast, strains capable of maintaining metabolic activity during stationary phase, tolerating increasing ethanol levels, and efficiently redirecting carbon flux toward fermentation products may outperform faster‐growing competitors in practical brewing systems [30].

The successful scale‐up of A2 to 100 L represents one of the most relevant outcomes of this study. Newly isolated environmental yeasts frequently perform adequately at flask scale but fail during pilot‐ or production‐scale fermentation due to changes in oxygen transfer, hydrostatic pressure, heat dissipation, and sedimentation dynamics. In the present work, A2 maintained a structured three‐phase fermentation profile and reached 3.8% ABV with 50.66% attenuation, showing an overall fermentation performance comparable with that of the commercial US‐05 strain. Although the attenuation was relatively low, this observation is likely related to the limited utilization of maltose and, potentially, maltotriose, as discussed above. This result is particularly significant because it demonstrates that a nondomesticated yeast originating from a nonbrewing environment can compete with an established industrial starter under realistic brewing conditions.

Comparable observations have been reported for other wild or nonconventional yeasts evaluated under pilot‐scale brewing conditions. Nikulin et al. isolated eight *Saccharomyces paradoxus* strains from oak trees in Finland and selected strain Sp3 for 40 L lager beer production. After adaptation, fermentation at 15°C was completed in 17 days, reaching 5.7% ABV in 15°P wort. Maltose consumption was as efficient as that of the reference lager strain, although no maltotriose utilization was detected. Sensory analysis indicated that the resulting beers were rated as equal to or better than the commercial controls, with full‐bodied character and a relatively clean flavor profile despite the presence of clove‐like 4‐vinyl guaiacol [31]. These results demonstrate that environmental yeasts can become technologically competitive when properly selected and conditioned. Likewise, Molinet and Cubillos reviewed multiple cases in which wild *S. cerevisiae* strains contributed to desirable industrial traits absent or limited in commercial starters. They highlighted that wild strains often show broader stress tolerance, increased production of aroma‐active metabolites such as isoamyl acetate and ethyl acetate, and higher genetic diversity than domesticated lineages. In addition, specific wild alleles have been linked to improved nitrogen consumption, glycerol production, freeze‐thaw tolerance, and adaptation to nutrient‐limited fermentations [9].

Hydrostatic stress has been reported as one of the most important challenges during scale‐up, as it can affect membrane fluidity, nutrient transport, and flocculation gene expression in *S. cerevisiae*. According to Abe, pressures as low as 10 MPa may already interfere with nutrient uptake, whereas pressures of 15–20 MPa can impair alcohol fermentation and pressures of 25–50 MPa alter membrane protein function by increasing membrane rigidity and modifying transporter activity [32]. These effects are particularly relevant in taller fermentation vessels, where yeast cells at the bottom are exposed to greater hydrostatic loads than those encountered in laboratory‐scale systems.

In the present work, the flocculation behavior of isolate A2 may have been influenced by this phenomenon, as reflected in the clarification differences observed between A2 and US‐05. The commercial strain exhibited stronger and faster biomass flocculation, consistent with the high sedimentation performance commonly selected during industrial domestication. In contrast, A2 remained in suspension for a longer period, resulting in higher transient turbidity during fermentation. This response may be linked to pressure‐mediated changes in membrane organization and cell wall physiology, since hydrostatic stress has been shown to induce transcriptional responses involving mannoproteins and cell envelope maintenance systems in yeast [32].

Despite this altered sedimentation pattern, A2 maintained satisfactory fermentative performance, reaching 3.8% ABV and 50.66% attenuation at 100 L scale. This indicates that any pressure‐associated effects were moderate and did not compromise metabolic activity. Moreover, the clarification difference disappeared after cold conditioning and bottling, indicating that the lower natural flocculation of A2 can be managed through standard cellar practices rather than representing a technological barrier. From a brewing perspective, moderate delayed flocculation may even be advantageous in some beer styles by prolonging yeast contact during maturation and favoring flavor development before final sedimentation.

The spectral changes observed after fermentation are consistent with the biochemical transformations that occur during brewing. Polshin et al. [22] reported that the FT‐IR fingerprint region is dominated by absorptions associated with carbohydrates and ethanol, making it particularly suitable for monitoring compositional changes during beer fermentation. Similarly, Grassi et al. [21] demonstrated that this region contains highly overlapped signals from fermentable sugars and ethanol, making the assignment of individual bands difficult by direct visual inspection. However, progressive variations in band intensity accurately reflect the consumption of wort carbohydrates and the formation of fermentation products, with the most pronounced changes occurring in the 1200–950 cm^−1^ region, where characteristic absorptions of maltose, maltotriose, dextrins, and ethanol are concentrated. These findings support the use of FT‐IR as a rapid, nondestructive technique for monitoring beer fermentation and comparing the metabolic performance of different yeast strains. Accordingly, the differences observed between isolate A2 and the commercial strain US‐05 suggest subtle variations in the composition of fermentation‐derived compounds, reflecting differences in their metabolic activity.

The sensory data are particularly relevant because they demonstrate that strain selection translated into perceptible product differentiation. Beer fermented with A2 showed stronger phenolic and sweet attributes, whereas the control beer exhibited a more conventional bitter‐malty profile. The clove‐like notes reported by panelists strongly suggest formation of 4‐vinyl guaiacol derived from ferulic acid decarboxylation, which is associated with the POF+ phenotype mediated by *PAD1/FDC1* activity [33]. In mainstream American ale styles such character may be considered atypical, but in wheat beers, Belgian ales, farmhouse beers, and specialty craft products it is often desirable. Therefore, the sensory profile of A2 should be interpreted as style‐dependent differentiation rather than a defect [34].

The present results indicate that nondomesticated *S. cerevisiae* strains isolated from traditional fermented beverages can exhibit fermentation performance comparable with that of commercial brewing yeasts while producing distinct sensory characteristics. These findings support the potential of traditional fermented products as valuable sources of yeast diversity for the selection of strains with brewing applications.

## 5. Conclusions

This study demonstrated that spontaneous nonbrewing fermentation systems are valuable sources of functional *S. cerevisiae* diversity with potential applications in brewing biotechnology. Among the isolates evaluated, strain A2 showed the best overall performance, combining efficient sugar utilization, satisfactory ethanol production, and stable behavior during pilot‐scale fermentation. Importantly, its fermentation performance was comparable with that of a commercial brewing yeast, despite originating from an environment unrelated to beer production. In addition, A2 generated a differentiated sensory profile characterized by phenolic and sweet notes while maintaining overall product acceptability.

The successful scale‐up of A2 indicates that nondomesticated yeasts can be incorporated into industrial brewing processes without compromising fermentation efficiency or product quality. These findings position isolate A2 as a promising alternative starter culture for draft beer production and as a candidate for future domestication programs based on naturally occurring microbial resources. Further studies should include whole‐genome sequencing, volatile compound profiling, repeated repitching trials, and validation in different beer styles to determine its full industrial potential.

## Author Contributions

All authors contributed to the study conception and design. Material preparation, data collection and analysis were performed by Raúl Alan Velázquez‐García, Erika Yanet Tapia‐García, Jorge Oscar Rivera‐Hernández, and Fortunata Santoyo‐Tepole. The first draft of the manuscript was written by Raúl Alan Velázquez‐García. Conceptualization, validation, supervision, writing—review and editing, and resources were contributed by Erika Yanet Tapia‐García, Guadalupe Guerra‐Sánchez, and Dario Rafael Olicón‐Hernández. Project administration was performed by Dario Rafael Olicón‐Hernández.

## Funding

This study was supported by the Secretaría de Investigación y Posgrado, Instituto Politécnico Nacional (10.13039/501100007161; 2391, 20251269, 20260257, 20251139, 20260225, 2024158).

## Disclosure

All authors commented on previous versions of the manuscript and read and approved the final manuscript.

## Conflicts of Interest

The authors declare no conflicts of interest.

## Supporting information


**Supporting Information** Additional supporting information can be found online in the Supporting Information section. The following supporting information is available: Figure S1: Mean intensity scores of the 25 sensory attributes evaluated in beers produced using the pulque‐derived *Saccharomyces cerevisiae* isolate A2 and the commercial brewing strain US‐05. The figure compares the sensory profiles of both beers, highlighting statistically significant differences in perceived sweetness and phenolic flavor. Error bars represent the standard error of the mean, and asterisks (^*^) indicate statistically significant differences.

## Data Availability

The data that support the findings of this study are openly available in National Center for Biotechnology Information at https://www.ncbi.nlm.nih.gov/, Reference Number PZ320974, PZ320973 and PZ320981.
