# Supplementary material for: Draft Beer Production Using a Nondomesticated Saccharomyces cerevisiae: Pilot‐Scale Comparison With a Commercial Brewing Yeast and Sensory Differentiation
Source: Int J Food Sci. 2026 Nov 10;2026:9534073. doi: 10.1155/ijfo/9534073 (PMC13749073; doi:10.1155/ijfo/9534073)
Supplement: Supplementary file 1 [file IJFO-2026-9534073-s001.docx]

Supplementary material

**Fermentation performance of pulque-derived yeasts in draft beer production: screening, scale-Up, and sensory profiling**

Raúl Alan Velázquez-García^a^, Erika Yanet Tapia-García^b^, Fortunata Santoyo-Tepole^c^, Jorge Oscar Rivera-Hernández^a^, Guadalupe Guerra-Sánchez^a*^, Dario Rafael Olicón-Hernández^a*^

**Figure S1. Mean intensity scores of the 25 sensory attributes evaluated for beers fermented with isolate A2 and the commercial strain US-05.** Overall, both beers exhibited similar sensory profiles, with significant differences detected in only two attributes. Beer produced with isolate A2 showed higher perceived sweetness and a more pronounced phenolic flavor, whereas the US-05 control exhibited a more neutral sensory profile. Error bars represent the standard error of the mean. Asterisks (*) indicate statistically significant differences in sweetness and phenolic flavor; no significant differences were observed for the remaining attributes.
